# 基于后修饰氯功能化二维共价有机骨架的微固相萃取法富集有机氯农药

**DOI:** 10.3724/SP.J.1123.2026.04026

**Published:** 2026-11-08

**Authors:** Haili ZHAO, Xiaohui DENG, Wenhao WANG, Zhilan WANG, Pengxin ZHOU, Shuhui HUO

**Affiliations:** 西北师范大学化学化工学院，高原交汇区水资源安全与水环境保护教育部重点实验室，甘肃 兰州 730070; Key Laboratory of Water Security and Water Environment Protection in Plateau Intersection （NWNU），Ministry of Education，College of Chemistry and Chemical Engineering，Northwest Normal University，Lanzhou 730070，China

**Keywords:** 共价有机骨架, 微固相萃取, 有机氯农药, 后修饰策略, covalent organic frameworks （COFs）, micro-solid-phase extraction （μ-SPE）, organochlorine pesticides （OCPs）, post-synthetic modification

## Abstract

有机氯农药（OCPs）是典型持久性有机污染物，具有高毒性、生物蓄积性与难降解性，严重威胁生态环境与人体健康。复杂水体基质中痕量OCPs富集困难，商用吸附材料选择性有限，传统前处理有机溶剂消耗量大、富集效率偏低。本研究以毛玻璃片为载体，负载氯功能化共价有机骨架材料（Cl-COF），构建微固相萃取（μ-SPE）体系，用于水样中OCPs的高效富集。利用环加成反应在共价有机骨架上引入富含卤素的活性位点，结合红外光谱、X射线衍射、热重分析、X射线光电子能谱、孔结构分析及水接触角测试完成材料的系统表征。结果表明，氯官能化后修饰过程未破坏COF晶体骨架结构。Cl-COF热解温度提升至363 ℃，耐酸碱耐溶剂性优异，氯原子占比为9.77%，水接触角由99.54°升至127.10°，疏水性大幅提升。尽管比表面积与孔径略有下降，但仍保有充足吸附位点。疏水作用、卤键作用、*π-π*堆积作用协同驱动OCPs萃取富集。将Cl-COF用于涂层，与气相色谱-电子捕获检测器（GC-ECD）联用，建立μ-SPE-GC-ECD检测方法。该方法中5种OCPs目标物在各自线性范围内线性关系良好，检出限为0.20~0.42 ng/L，富集因子为891~1 308；兰州段黄河水和菜田浇灌水的加标回收率分别为89.2%~104.0%和89.0%~105.5%，相对标准偏差分别为1.7%~11.3%和2.4%~12.8%，具备良好的准确度、精密度与基质抗干扰能力。Cl-COF与玻片基底结合牢固，经30次吸附-解吸循环后萃取性能无明显衰减，稳定性与再生性能良好。相较于已报道检测方法，本方法综合萃取性能更佳，可为水环境中OCPs常态化监测提供技术支撑。

## Abstract (en)

Organochlorine pesticides （OCPs），typical persistent organic pollutants （POPs），are toxic，bioaccumulative and recalcitrant，threatening ecological safety and human health. Enriching trace OCPs from complex water matrices remains challenging due to the limited selectivity of commercial adsorbents and the low efficiency of traditional solvent-intensive pretreatment techniques. In this work，frosted glass slides were adopted as supporting substrates to immobilize post-modified chloro-functionalized covalent organic frameworks （Cl-COF） for micro-solid-phase extraction （μ-SPE） of OCPs. Chlorine atoms were successfully introduced into two-dimensional covalent organic frameworks via a cycloaddition reaction to synthesize Cl-COF with abundant halogen-active sites. FT-IR，XRD，TGA，XPS，nitrogen adsorption-desorption，and water contact angle measurements were used for systematic material characterization. FT-IR spectra confirmed the successful Schiff-base polymerization and grafting of chlorine groups. XRD patterns demonstrated that post-modification did not damage the crystalline framework of the COF，and the prepared material exhibited outstanding resistance to acids，alkalis and organic solvents，with its thermal stability improved from 317 ℃ to 363 ℃. Although the specific surface area and pore size slightly decreased after functionalization，abundant adsorption sites were still retained for target capture. XPS analysis revealed a surface chlorine atomic ratio of 9.77%，further verifying the successful fabrication of Cl-COF. Furthermore，the water contact angle increased from 99.54° to 127.10°，indicating a remarkably enhanced hydrophobicity of the modified material. Systematic characterizations reveal that the obtained Cl-COF possesses a large specific surface area，excellent chemical stability and hydrophobicity，and exhibits prominent extraction and adsorption performance toward OCPs. Mechanism investigations verify that the synergistic effects of hydrophobic interaction，halogen bonding between chlorine sites and OCP molecules，as well as interlayer *π*-*π* stacking interaction dominate the extraction and enrichment of trace OCPs on Cl-COF. The as-fabricated Cl-COF coating was combined with gas chromatography equipped with an electron capture detector （GC-ECD） to establish a novel μ-SPE-GC-ECD analytical method. Methodological validation was carried out to evaluate the analytical performance of the established method. The results demonstrated that the proposed method achieves a wide linear range for the five OCPs，with a low limit of detection of 0.20–0.42 ng/L and high enrichment factors ranging from 891 to 1 308. Satisfactory recoveries of 89.2%–104.0% and 89.0%–105.5% were obtained in two types of real water samples，and the relative standard deviations （RSDs） ranged from 1.7%–11.3% and 2.4%–12.8%，proving good accuracy，precision and anti-interference ability for complex aqueous matrices. Moreover，the composite material adheres firmly to the glass slide substrate without delamination under repeated elution and washing procedures，and no obvious decay in extraction capacity is observed after 30 consecutive adsorption-desorption cycles，demonstrating its superior structural stability and outstanding reusability. Compared with previously reported μ-SPE methods for OCPs determination，this proposed strategy exhibits better comprehensive extraction performance. This study provides feasible technical support for routine safety supervision and rapid detection of OCPs in aquatic environments.

有机氯农药（organochlorine pesticides，OCPs）凭借其高效的杀虫活性，曾在农业生产中得到广泛应用，通常被用于控制农作物病虫害^［1，2］^。然而，由于其具有持久性、生物蓄积性，OCPs对人体健康和生态系统构成严重威胁，已引发全球广泛关注^［3，4］^。研究表明，喷洒的OCPs中仅有约0.1%作用于靶标害虫，其余99.9%则通过地表径流、喷雾漂移和渗透等途径进入周边环境^［5］^。尽管《斯德哥尔摩公约》已明确禁止OCPs的生产和使用，但部分地区仍存在非法使用现象，导致其残留物持续存在于环境和食品中^［6］^。人体长期接触OCPs可能引发癌症、生殖功能障碍、神经系统及呼吸系统疾病，并影响智力发育，因此被列为典型的持久性有机污染物（POPs）之一^［7］^。当前，针对OCPs及其代谢物的定量分析，主流依托色谱及联用检测方法，包括气相色谱（GC）、高效液相色谱（HPLC）、气相色谱-质谱（GC-MS）和液相色谱-质谱（LC-MS）等仪器分析技术^［8，9］^。受限于样品基质复杂、目标物含量低微，样品前处理环节对整个分析流程的准确性与灵敏度起到决定性作用。微固相萃取（μ-SPE）作为一种绿色、高效的样品前处理技术，通过将少量吸附剂固定于固体载体上，利用目标物与吸附剂之间的分配平衡实现富集。然而，由于实际水体中常同时存在多类污染物，传统针对单一类别污染物的检测方法已难以胜任日益繁重的水质监测任务。因此开发新型吸附材料并建立灵敏、准确的分析方法，对环境与食品中痕量OCPs的检测具有重要意义。

共价有机骨架材料（covalent organic frameworks，COFs）作为一类新兴多孔晶体聚合物，因其高比表面积、优异的孔隙结构及良好的化学稳定性，在吸附分离领域展现出广阔应用前景^［10-17］^。特别是，通过引入特定官能团的后合成修饰策略（post-synthetic modification，PSM）可显著增强COFs对有机污染物的特异性富集能力^［18］^。例如，Hu等^［19］^利用氟原子的疏水性和强电负性特点，引入氟原子，成功制备氟化共价有机骨架，利用卤素键作用和*π-π*堆积作用，实现了对5种苯并酰脲类杀虫剂的高效富集。Wu等^［20］^采用原位制备方法，利用不锈钢丝作为载体合成了共价有机框架CF-COF，实现了对多氯联苯的高效富集。

尽管如此，目前基于COF的微固相萃取（μ-SPE）装置用于水体中OCPs的富集研究仍较少，且相关吸附剂的固载方式有待进一步简化。本文通过后修饰法在二维TAPT-PTA-COF骨架上引入氯取代基，制备了氯功能化的TAPT-PTA-COF骨架，即Cl-COF。将Cl-COF固载于玻片表面，构建了基于Cl-COF的μ-SPE装置，用于OCPs的高灵敏富集。通过系统优化萃取条件，并联用气相色谱-电子捕获检测器（GC-ECD）技术，构建了针对菜田浇灌水和黄河水中痕量OCPs的分析方法。本工作为环境中持久性有机污染物的高效分离富集及检测提供了新思路。

## 1 实验部分

### 1.1 仪器及试剂

Agilent GC-6890型气相色谱仪（Agilent公司，美国），FTS-3000型傅里叶变换红外光谱仪（FT-IR，Digilab公司，美国），D/max-2200型X射线衍射仪（XRD，Rigaku公司，日本），TGA-4000型热重分析仪（TGA，Anton Paar公司，美国），Autosorb iQ型全自动比表面积与孔径分析仪（Quantachrome公司，美国），K-Alpha型X射线光电子能谱仪（XPS，Thermo Fisher Scientific公司，美国）。

2，4，6-三（4-氨基苯基）-1，3，5-三嗪（TAPT）、对苯二甲醛（PTA）、4-氯苯乙炔、四氯苯醌、三氟化硼乙醚（BF_3_·OEt_2_）、四氢呋喃（THF）（均为分析纯，购自上海泰坦科技有限公司）；1，4-二氧六环、均三甲苯（均为分析纯，购自上海阿达玛斯试剂公司）；5种有机氯农药标准品（购自英国LGC集团）：1，1-二氯-2-（邻氯苯基）-2-（对氯苯基）乙烷（2，4′-DDD）、1，1，1-三氯-2-（邻氯苯基）-2-（对氯苯基）乙烷（2，4′-DDT）、1，1-二氯-2-（邻氯苯基）-2-（对氯苯基）乙烯（2，4′-DDE）、1，4，5，6，7，8，8-七氯-2，3-环氧-3a，4，7，7a-四氢-4，7-甲基茚（环氧七氯）、1，4，5，6，7，8，8-七氯-3a，4，7，7a-四氢-4，7-甲基茚（七氯）。实际水样采集自兰州市黄河干流及周边农田灌溉渠。

### 1.2 标准溶液配制

精确称取5种有机氯农药标准品，用甲醇配制成标准储备液（10.0 mg/L）。使用蒸馏水逐级稀释，配制0.10、0.20、0.50、1.0、10.0、50.0、100、1 000 ng/L工作溶液。

### 1.3 GC-ECD条件

采用GC-6890气相色谱仪，配备^63^Ni源的电子捕获检测器（ECD），以SE-30毛细管柱（30 m×0.25 mm×0.25 μm）进行分离。载气为高纯氮气（纯度99.99%），采用无分流进样方式。程序升温条件：初始温度100 ℃，保持2 min；以60 ℃/min升温至160 ℃；接着以10 ℃/min升温至240 ℃保持5 min；最后以50 ℃/min升温至280 ℃，保持5 min。进样口温度280 ℃，检测器温度300 ℃，进样体积为1 µL。

### 1.4 TAPT-PTA-COF和Cl-COF的制备

将0.15 mmol的PTA与0.10 mmol的TAPT溶解于10 mL均三甲苯-1，4-二氧六环（1∶9，*V/V*）的混合溶液中，超声使其分散均匀，转移至高压反应釜中。随后，加入0.6 mL 6 mol/L的冰醋酸作为催化剂，在120 ℃条件下反应72 h。待反应体系冷却后，产物经THF多次洗涤后真空干燥，得到黄色粉末TAPT-PTA-COF。

依次称取40.0 mg TAPT-PTA-COF、120.0 mg 4-氯苯乙炔及80.0 mg四氯苯醌，置于圆底烧瓶中，并依次加入40 μL BF_3_·OEt_2_和10 mL甲苯。在氮气保护下，于110 ℃搅拌反应24 h。8 000 r/min高速离心10 min分离得到固体产物，用THF洗涤后，再加入饱和Na_2_CO_3_溶液进行淬灭处理。采用THF索氏提取12 h后，60 ℃真空干燥，得到Cl-COF材料。合成路线示于图1。

**图1:**
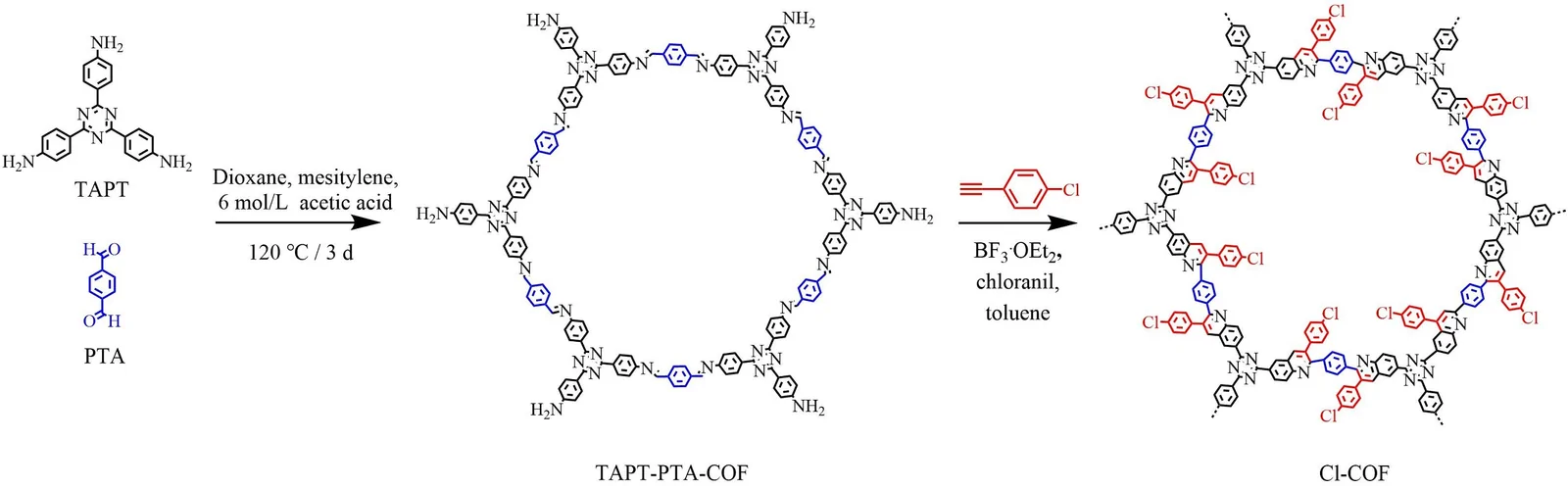
TAPT-PTA-COF和Cl-COF的合成路线 TAPT： 2，4，6-tris（4-aminophenyl）-1，3，5-triazine； PTA： terephthalaldehyde； Cl-COF： chloro-functionalized TAPT-PTA-COF.

### 1.5 **μ**-SPE玻片的制备

将规格1 cm×1.5 cm长方形毛玻璃片置于6 mol/L NaOH溶液中浸泡蚀刻2 h，以此提升玻片表面粗糙度；蚀刻结束后，采用去离子水冲洗玻片，置于室温晾干待用。准确称取15.0 mg Cl-COF粉末，加入2 mL环氧树脂胶充分混合均匀，将混合液均匀涂覆于预处理后的玻片表面，室温晾干后，置于烘箱80 ℃下固化12 h，制得Cl-COF功能涂层修饰玻片。此外，保持玻片预处理、环氧树脂涂布、热固化全部实验条件一致，仅将Cl-COF粉末替换为等量TAPT-PTA-COF粉末，制备得到无氯修饰的TAPT-PTA-COF玻片，作为空白对照样品。

### 1.6 **μ**-SPE步骤

实际水样以8 000 r/min转速离心8 min，去除水体泥沙及悬浮固体颗粒物；吸取上层清液，经水系微孔过滤膜过滤处理，收集滤液，4 ℃避光冷藏备用。量取10 mL处理后的水样置于玻璃瓶内，放入Cl-COF玻片，密封玻璃瓶后，60 ℃恒温萃取15 min。萃取结束后取出玻片，转至洗脱瓶中，加入3 mL乙腈对玻片表面富集目标物进行脱附处理。待目标物完全脱附后收集洗脱液，氮气流吹干，采用100 μL乙腈复溶，经0.22 μm有机系滤膜过滤后，采用GC-ECD进样分析。水体不同浓度加标样品的微固相萃取及后续前处理操作步骤均与上述流程一致。样品前处理与仪器检测流程如图2所示。

**图2:**
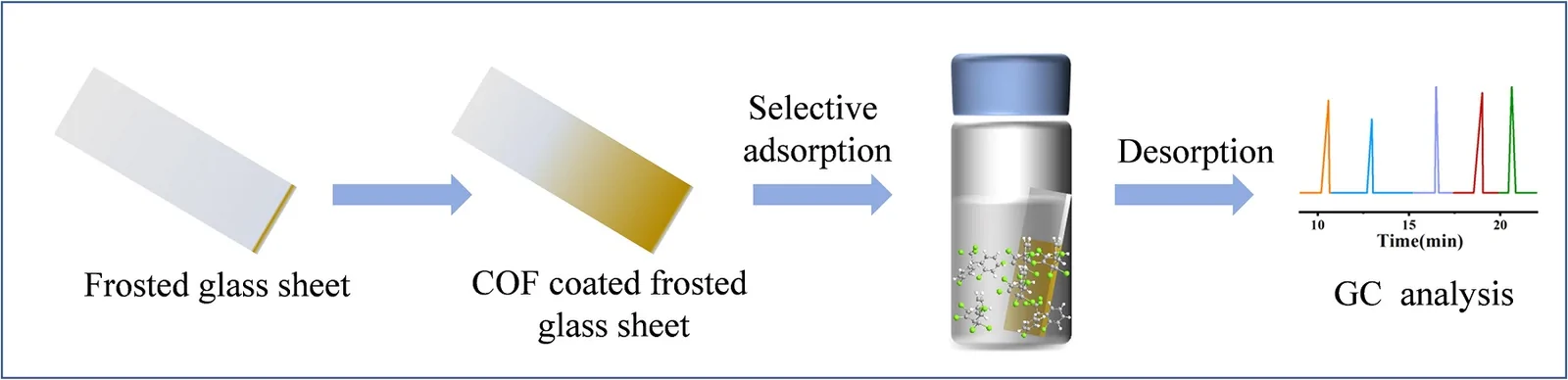
从水样中提取OCPs的μ-SPE流程图 μ-SPE： micro-solid phase extraction； OCPs： organochlorine pesticides.

## 2 结果与讨论

### 2.1 Cl-COF材料的表征

采用FT-IR表征单体和两种COF材料的官能团变化。图3a显示，TAPT单体在3 200~3 500 cm⁻¹处的氨基特征伸缩振动峰在聚合后显著减弱，证明TAPT单体中的氨基与PTA中的醛基成功发生席夫碱缩合反应。对于Cl-COF，在656 cm⁻¹处出现了C-Cl键的特征伸缩振动峰，证实氯官能团成功引入COF骨架。

**图3:**
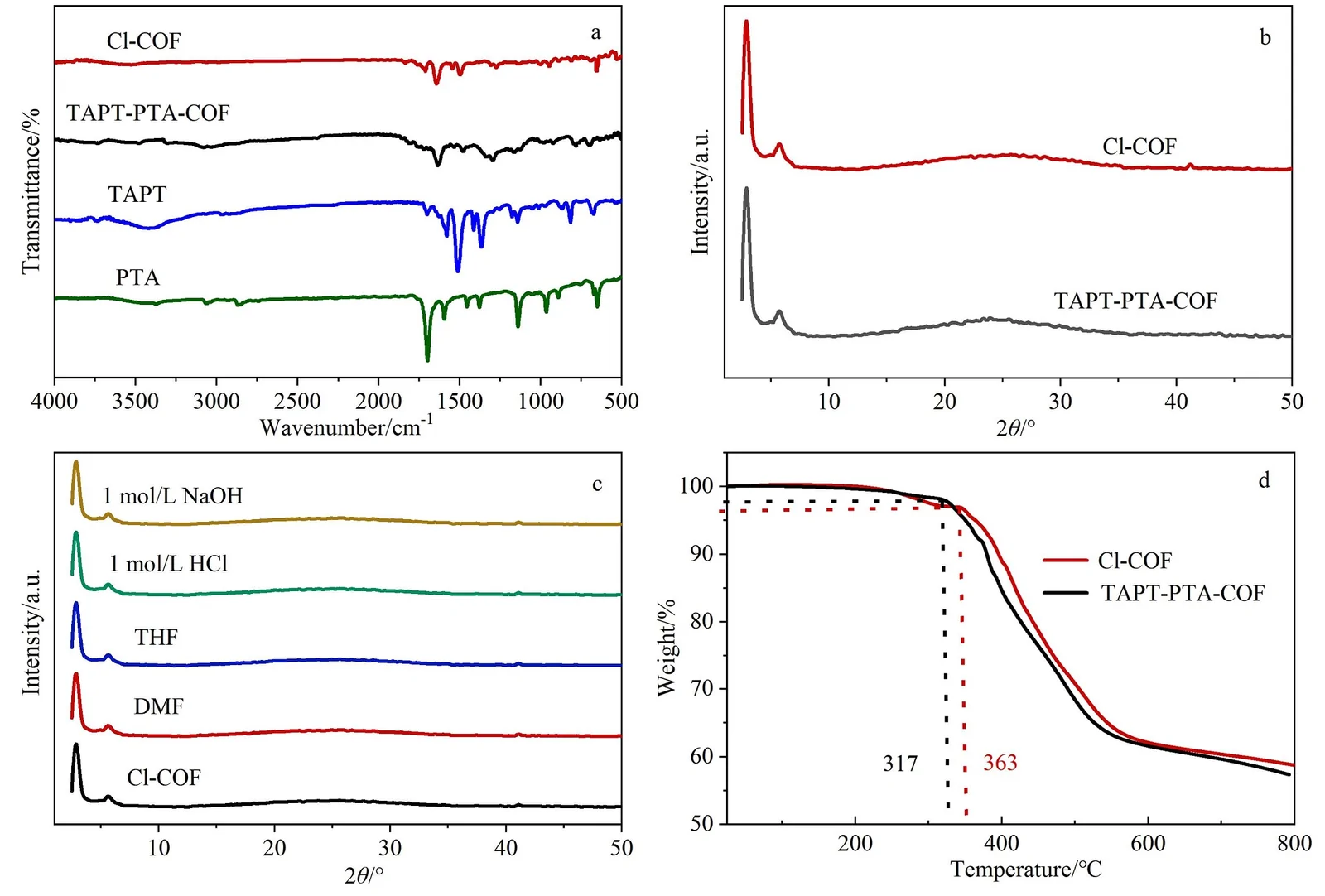
TAPT-PTA-COF和Cl-COF的结构与稳定性表征 a. FT-IR spectra； b. XRD patterns； c. XRD patterns of Cl-COF after treatment with various solvents； d. TGA curves.

利用XRD考察了两种COF的晶体结构（图3b），TAPT-PTA-COF在2*θ*=2.8°处呈现一个强衍射峰，归属于（100）晶面；2*θ*=26.6°处呈现一宽衍射峰，该现象与现有结论一致^［21］^，印证TAPT-PTA-COF层间存在*π*-*π*堆叠结构。经后修饰合成得到的Cl-COF，其XRD谱图与TAPT-PTA-COF基本一致，表明氯官能化过程未破坏COF的原有晶型结构。为评估Cl-COF在酸碱和不同溶剂环境中的稳定性，将其分别浸泡于1 mol/L NaOH、1 mol/L HCl、DMF、THF中72 h。通过XRD结构比较发现Cl-COF的衍射峰未发生明显变化（图3c），说明该材料在极端酸碱条件下和DMF、THF中均能保持很好的稳定性，具有优异的化学稳定性。

借助TGA考察TAPT-PTA-COF及其后修饰产物Cl-COF在20~800 ℃范围内的热稳定性，结果如图3d所示。TAPT-PTA-COF在317 ℃时开始出现明显失重，材料内部骨架结构逐步塌陷；相比之下，Cl-COF热稳定性明显提升，其骨架结构可在363 ℃以下保持完整，温度超过363 ℃后才发生缓慢失重。综合分析可知，4-氯苯乙炔后修饰改性不会破坏母体TAPT-PTA-COF骨架结构，引入的氯官能团可有效提升材料耐高温能力与整体热稳定性。

在温度为‒196.15 ℃、相对压力（relative pressure，*p*/*p*_0_）0~1.0条件下，测定TAPT-PTA-COF和Cl-COF的氮气吸附-脱附等温线（图4a、4b）。TAPT-PTA-COF呈典型Ⅰ型吸附等温线，BET比表面积为904 m^2^/g，平均孔径约1.7 nm。经4-氯苯乙炔后修饰的Cl-COF，其比表面积降至723 m^2^/g，孔径缩小至1.2 nm。该结果表明，引入的氯苯乙炔基团占据了部分孔道空间，导致材料孔径和比表面积小幅下降，但Cl-COF仍具备丰富的孔隙结构，能够为吸附OCPs提供充足的吸附位点。

**图4:**
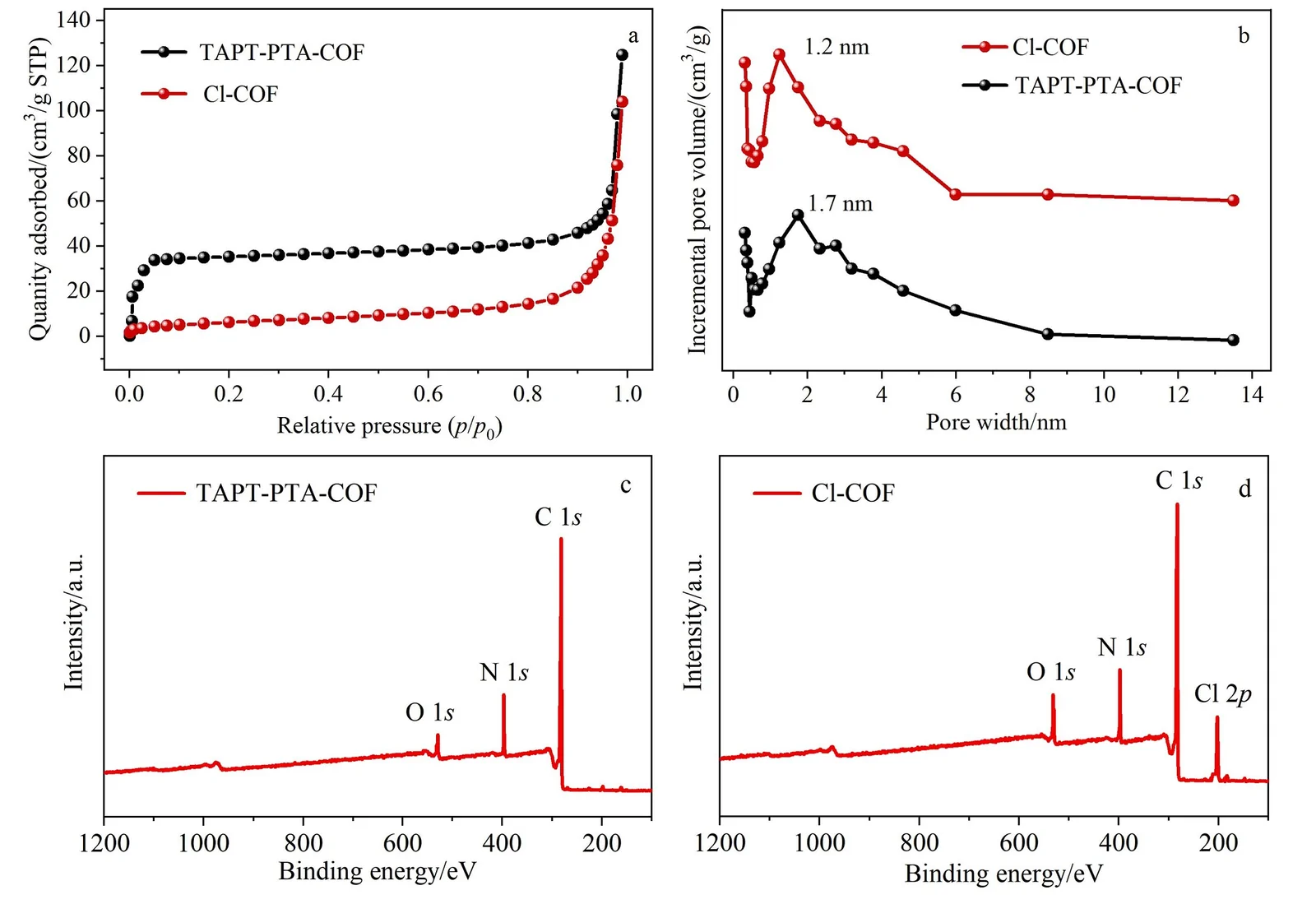
TAPT-PTA-COF和Cl-COF的（a）氮气吸附-脱附等温线、（b）孔径分析和（c、d）XPS全谱分析 XPS： X-ray photoelectron spectroscopy.

采用XPS分析TAPT-PTA-COF与Cl-COF的化学元素组成。XPS全谱扫描结果显示，TAPT-PTA-COF仅出现C、N、O 3种元素特征吸收峰，与其理论元素组成相符（图4c）。经4-氯苯乙炔后修饰改性后，Cl-COF在保留C、N、O特征峰的基础上，于结合能200 eV处出现显著Cl 2*p*特征吸收峰（图4d），证实氯元素成功引入COF中。元素定量分析结果表明，Cl-COF表面氯元素原子占比为9.77%。综上，本研究通过后合成修饰策略，成功制备氯功能化改性Cl-COF目标材料。

本研究通过静态接触角测试表征TAPT-PTA-COF和Cl-COF 2种材料的表面润湿性，以此评价2种材料对疏水性OCPs的富集能力。接触角测试结果如图5a、5b所示，TAPT-PTA-COF的静态接触角为99.54°，说明基底COF本身具备中等疏水性；经4-氯苯乙炔修饰后得到Cl-COF，材料接触角提升至127.10°，疏水性得到显著增强。疏水性提升源于疏水性芳基氯官能团的成功引入，该官能团可降低材料表面自由能，增强表面对水分子的排斥作用。优异的表面疏水性能使Cl-COF可通过疏水相互作用高效结合水体中的痕量OCPs，实现对目标有机氯农药的高效富集。

**图5:**
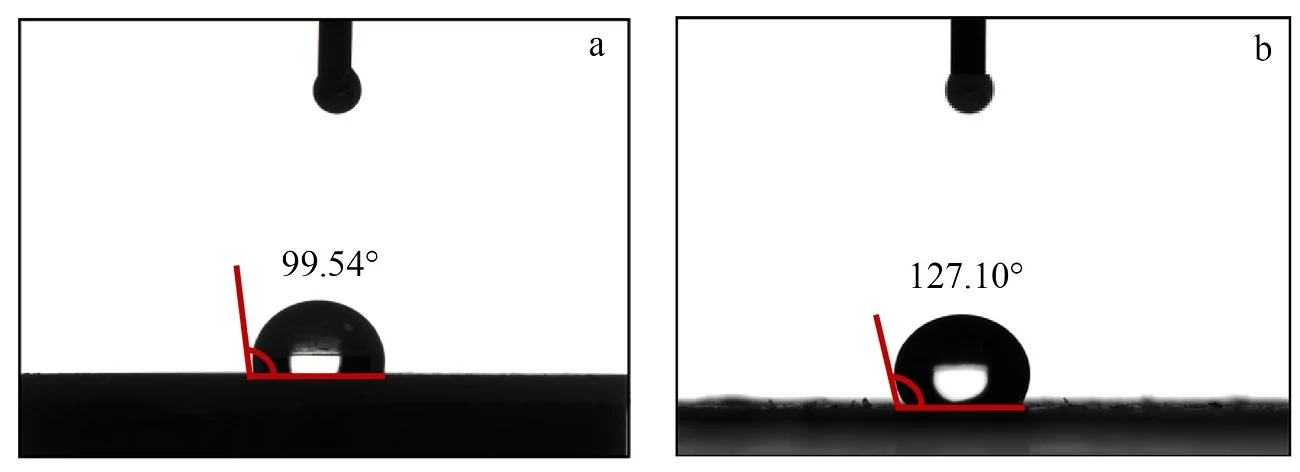
（a）TAPT-PTA-COF和（b）Cl-COF的静态接触角测试图

### 2.2 微固相萃取条件优化

为获得最佳萃取性能，本研究以Cl-COF作为微固相萃取涂层，采用GC-ECD对目标物进行检测，以色谱峰峰面积作为评价指标，来评估Cl-COF对5种OCPs的萃取效率。实验考察了不同氯含量COF、吸附剂添加量、离子强度、萃取时间与温度、解吸溶剂类型、洗脱液体积及涂层复用性等参数对Cl-COF萃取效果的影响。

为考察氯修饰含量对萃取性能的影响，在固定TAPT-PTA-COF用量40 mg的条件下，调节4-氯苯乙炔的质量，使其与TAPT-PTA-COF的质量比分别为1∶1、1∶2、1∶3、2∶1及3∶1，制备得到一系列不同氯功能化程度的复合材料，依次记为Cl-COF-1~5。由图6可知，Cl-COF-3对5种OCPs的富集效果最佳；相比之下，未修饰的TAPT-PTA-COF萃取能力最弱。上述结果证实，氯官能团的引入可显著提升材料对OCPs的富集能力。基于以上优化结果，后续实验选用40 mg TAPT-PTA-COF和120 mg 4-氯苯乙炔制备的Cl-COF作为μ-SPE吸附剂。

**图6:**
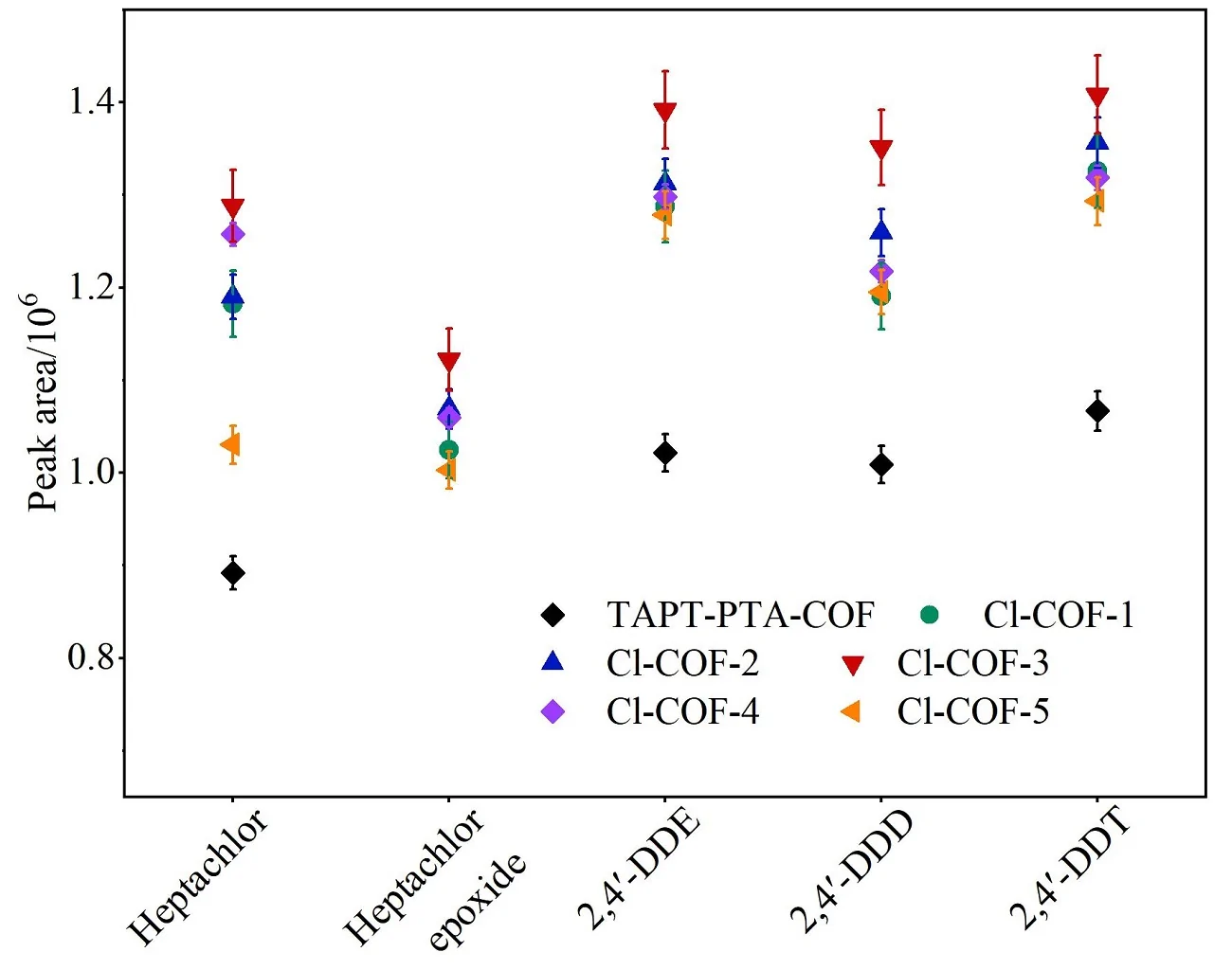
不同4-氯苯乙炔修饰的TAPT-PTA-COF的萃取性能对比（*n*=3） Cl-COF-1‒5 corresponds to mass ratios of 4-chlorophenylacetylene to TAPT-PTA-COF （40 mg） of 1∶1，1∶2，1∶3，2∶1 and 3∶1，respectively. 2，4′-DDE： 1，1-dichloro-2-（2-chlorophenyl）-2-（4-chlorophenyl）ethene； 2，4′-DDD： 1，1-dichloro-2-（2-chlorophenyl）-2-（4-chlorophenyl）ethane； 2，4′-DDT： 1，1，1-trichloro-2-（2-chlorophenyl）-2-（4-chlorophenyl）ethane.

吸附剂用量决定了可供利用的活性位点总数。本实验中固定萃取温度50 ℃、甲醇2 mL、NaCl加入量0.1 g、萃取时间15 min，分别考察5、10、15、20、25、30 mg Cl-COF对OCPs萃取效率的影响。如图7a所示，吸附剂用量在5~15 mg范围内增加时，OCPs峰面积相应增加，说明有效吸附位点增多。当吸附剂用量超过15 mg后，峰面积基本持平，趋于稳定，表明吸附位点冗余，故15 mg即可满足完全萃取需求，选为最佳用量。

**图7:**
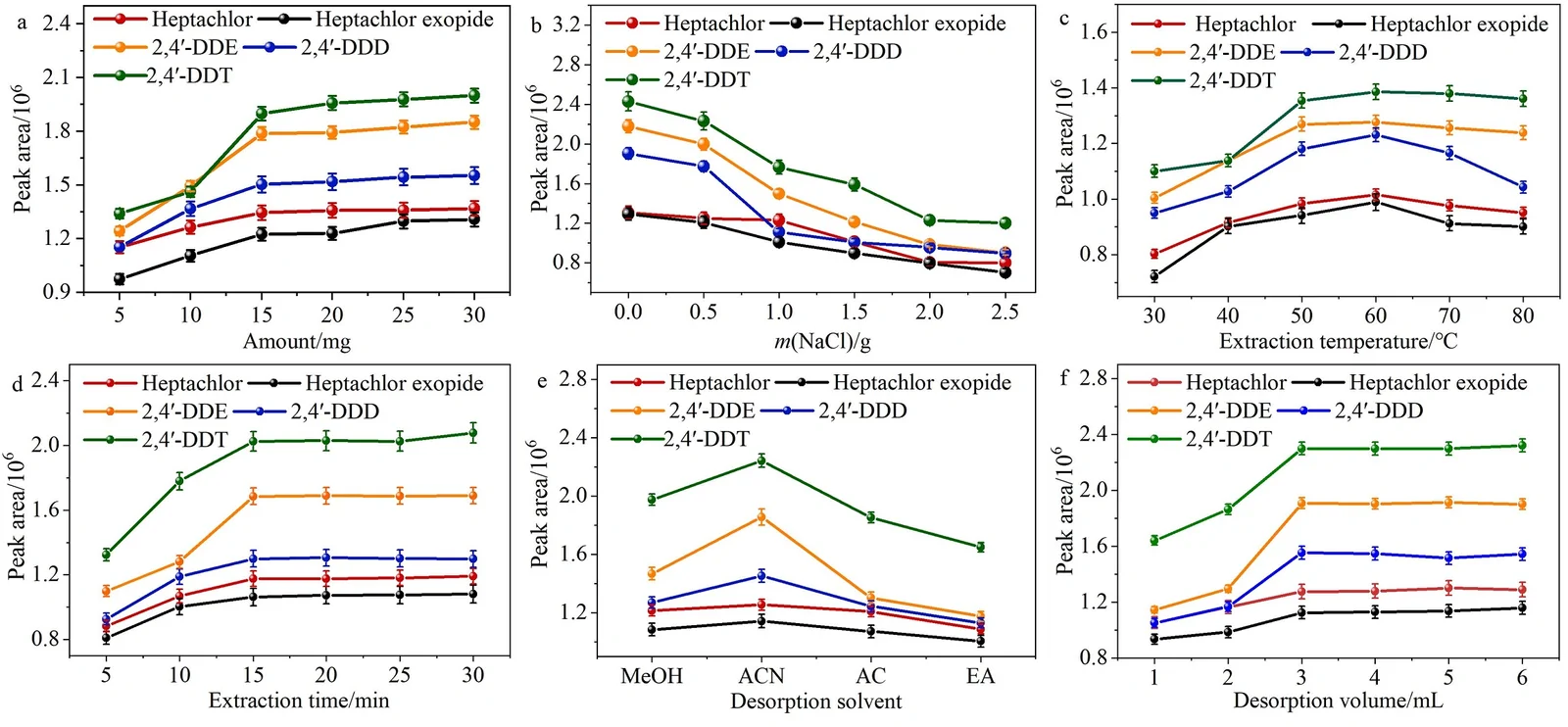
不同萃取条件对5种OCPs萃取效率的影响（*n*=3） a. Cl-COF dosage； b. ionic strength； c. extraction temperature； d. extraction time variation； e. desorption solvent； f. eluent volume. MeOH： methanol； ACN： acetonitrile； AC： acetone； EA： ethyl acetate.

在μ-SPE过程中，离子强度可通过盐析效应改变目标物在固液两相的分配系数，直接影响萃取效果。基于上述最优条件，本研究探究了离子强度对5种OCPs萃取效果的影响：在10 mL水样中分别加入0、0.5、1.0、1.5、2.0和2.5 g NaCl（图7b）。实验结果表明，随着添加NaCl量的升高，5种OCPs的色谱峰面积逐渐降低。因此，后续实验均不外加氯化钠调节离子强度。

萃取温度对μ-SPE过程存在双重效应，适当升高温度可加快传质速率，缩短萃取达到平衡所需的时间；温度过高则会降低两相分配系数，弱化萃取富集效果。因此，本研究在30~80 ℃的温度范围内考察了萃取温度对OCPs萃取效率的影响。如图7c所示，当萃取温度从30 ℃升至60 ℃时，目标物色谱峰面积显著增加，并在60 ℃时达到峰值，继续升温至80 ℃后，峰面积呈明显下降趋势。该现象表明，60 ℃条件下体系的传质过程可达到最佳平衡，过高的温度会破坏目标物与Cl-COF涂层之间的分配平衡，导致萃取性能下降。因此，本研究选取60 ℃作为最优萃取温度。

萃取时间是影响μ-SPE对OCPs萃取效果的重要因素。如图7d所示，当萃取时间由5 min延长至15 min时，目标物峰面积随时间延长显著增大；继续延长萃取时间，峰面积不再发生明显变化并趋于平稳，表明该萃取体系在15 min时即可达到吸附平衡，因此本研究选取15 min作为后续实验的最优萃取时间。

本研究对比考察了甲醇、乙腈、丙酮、乙酸乙酯4种有机溶剂对OCPs的洗脱性能（图7e）。实验结果表明，乙腈凭借适宜的极性与“类溶解”特性^［22］^，展现出最优的洗脱能力，5种OCPs的峰面积均达到最大值，解吸效果显著优于其他溶剂。因此，本实验选用乙腈作为μ-SPE体系的最优解吸溶剂。在确定最优解吸溶剂的基础上，进一步探究了乙腈洗脱体积对萃取效果的影响（图7f）。结果显示，随着乙腈用量的增加，OCPs洗脱效率逐步提升；当洗脱体积大于3 mL时，色谱峰面积基本保持恒定，表明3 mL乙腈可实现对材料表面目标分子的完全洗脱。综上，确定3 mL为最佳解吸溶剂体积。

### 2.3 方法学验证

在确定的最优μ-SPE萃取条件下，将制备的Cl-COF修饰玻片作为萃取介质对10 mL质量浓度分别为0.10、0.20、0.50、1.0、10.0、50.0、100、1 000 ng/L的5种OCPs混合标准溶液进行富集与分析，系统考察该分析方法的线性范围、检出限（LOD）、定量限（LOQ）、日内、日间及批间精密度（RSD）。结果如表1所示，环氧七氯、2，4′-DDD、2，4′-DDE的线性范围均为1.0~1 000 ng/L，七氯、2，4′-DDT线性范围为2.0~1 000 ng/L；各目标物色谱峰面积与对应浓度均呈良好线性关系，相关系数*R*^2^介于0.996 9~0.999 3。基于*S*/*N*=3和*S*/*N*=10分别计算LOD和LOQ，其值分别为0.20~0.42 ng/L和0.65~1.26 ng/L。

**表1:** 5种OCPs的线性范围、相关系数、检出限、定量限和精密度

Analyte	Linear range/（ng/L）	*R* ^2^	LOD/（ng/L）	LOQ/（ng/L）	Intra-day RSD/% （*n*=3）	Inter-day RSD/% （*n*=3）	Slide-to-slide RSD/% （*n*=3）	Enrichment factor
Heptachlor epoxide	1.0‒1000	0.9969	0.29	0.92	2.9	8.3	10.4	1202
Heptachlor	2.0‒1000	0.9974	0.34	1.12	4.3	5.4	9.0	1095
2，4′-DDD	1.0‒1000	0.9968	0.24	0.76	6.4	2.4	2.6	1265
2，4′-DDE	1.0‒1000	0.9993	0.20	0.65	3.6	7.5	8.2	1308
2，4′-DDT	2.0‒1000	0.9967	0.42	1.26	5.6	6.5	7.2	891

为评价Cl-COF对OCPs萃取检测方法的精密度，分别开展日内、日间及批间精密度试验。日内精密度为同一加标水样单日连续多次进样测定；日间精密度为相同加标样品连续多日分别测试。结果显示，5种OCPs日内RSD为2.9%~6.4%（*n*=3），日间RSD为2.4%~8.3%（*n*=3），表明本方法在灵敏度和精密度方面表现良好，能够满足实际水样中有机氯农药的检测需求。此外，在最优条件下，考察了不同批次制备的Cl-COF玻片对5种OCPs的萃取性能，批间RSD为2.6%~10.4%，表明Cl-COF修饰改性的玻片制备工艺稳定，材料批次重复性好。

为了评价Cl-COF对OCPs的富集性能，本研究采用富集因子（enrichment factor，EF）作为定量评价指标。其中，富集因子定义为μ-SPE富集后目标物的色谱峰面积与未富集直接检测的峰面积之比。实验结果显示，所制备的Cl-COF材料对5种OCPs的富集因子可达891~1 308，表明该Cl-COF萃取介质对目标有机氯农药具有优异的富集吸附能力。

将本研究制备的Cl-COF与现有文献报道的ZnO纳米颗粒、MIL-101、MnO_2_@CGO、rGO-amino-HNT@PT等多种μ-SPE吸附材料用于OCPs的萃取性能进行对比，结果如表2所示。相较于上述吸附介质，Cl-COF对OCPs展现出更优异的富集效果，其高效吸附源于多重作用协同：其一，Cl-COF具有较大比表面积和适宜孔隙结构，可为目标污染物OCPs提供丰富的吸附位点，有效提升材料的吸附容量；其二，骨架苯环与OCPs的共轭结构形成强*π*-*π*堆积作用，增强选择性吸附；其三，材料表面的强疏水性与疏水性OCPs产生疏水亲和作用；其四，氯改性引入的氯原子可与OCPs分子形成C-Cl…Cl-C卤键，相较于纯TAPT-PTA-COF，吸附性能显著提升。因此，丰富的吸附位点、*π*-*π*作用、疏水效应及卤键的多重协同效应，是Cl-COF高效捕获痕量OCPs的关键机理。

**表2:** 本研究与其他研究的对比

Method	Absorbent	Linear range/（ng/L）	LOD/（ng/L）	RSD/%	Ref.
μ-SPE-GC-MS	ZnO NPs	1000‒250000	190‒1640	2.3‒10.2	［^23^］
μ-SPE-GC-MS	MIL-101	50‒50000	2.5‒16	4.2‒11	［^24^］
μ-SPE -GC-MS	MnO_2_@CGO	100‒5000	5000‒12000	1.7‒8.7	［^25^］
d-μ-SPE-GC-MS	rGO-amino-HNT@PT	100‒2000	2‒13	6.1‒8.7	［^26^］
μ-SPE-GC-ECD	Cl-COF	1.0‒1000	0.20‒0.42	2.4‒10.4	this work

### 2.4 Cl-COF玻片的使用寿命研究

可重复利用性能是评判吸附材料实际应用潜力的核心指标。本研究通过多次循环萃取实验系统考察了Cl-COF功能化玻片的使用寿命与循环稳定性（图8）。实验结果表明，所制备的功能玻片在连续完成30次萃取循环后，对目标OCPs的萃取效率未产生明显衰减，说明Cl-COF功能涂层与玻片基底结合牢固，材料内部吸附骨架与活性位点能够长期稳定维持。当循环使用次数超过40次后，少量功能涂层出现轻微脱落，同时部分吸附活性位点会被体系中难以脱附的微量杂质逐步占据，双重因素共同作用导致材料的萃取效率产生10%~15%的小幅下降。

**图8:**
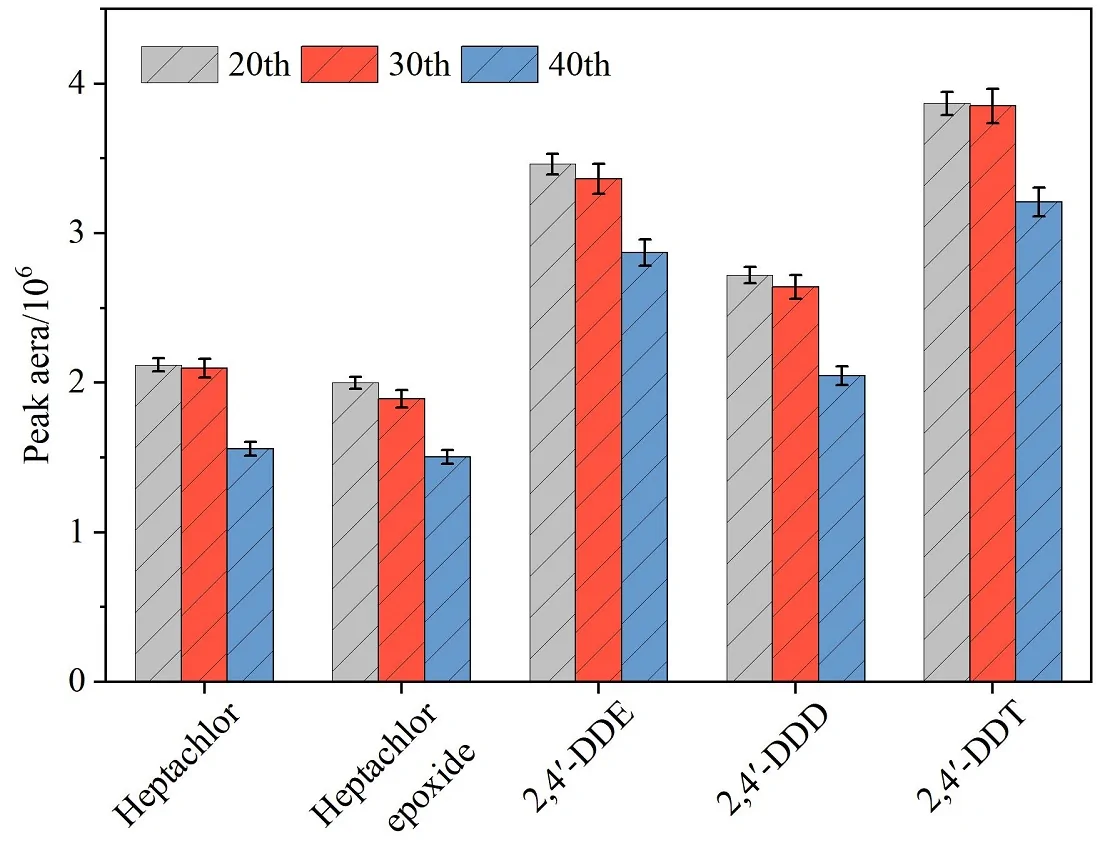
Cl-COF修饰玻片的循环使用性能（*n*=3）

### 2.5 实际样品分析

本研究建立了一种基于Cl-COF涂层玻片的微固相萃取方法，结合气相色谱-电子捕获检测器，用于测定黄河水及菜田灌溉水中的5种有机氯农药残留。通过空白水样加标回收实验，平行测定3次，验证了该方法的准确性和适用性。结果表明，两类空白水样中5种目标OCPs含量均低于方法的定量限。选取空白水样基质，在1.0、50和100 ng/L 3个加标水平下进行加标回收试验，每组样品采用玻片微固相萃取方式富集检测，平行测定3次。如表3所示，兰州段黄河水和菜田浇灌水中5种OCPs的萃取回收率分别为89.2%~104.0%和89.0%~105.5%，RSD分别为1.7%~11.3%和2.4%~12.8%。实际水样分析结果如图9所示，空白黄河水和菜田浇灌水样品中无基质杂质峰干扰；各浓度加标样品中的七氯、环氧七氯、2，4′-DDE、2，4′-DDD和2，4′-DDT的色谱峰峰形完整，组分分离度良好。由上述结果可知，本方法抗基质干扰能力强、检测准确度与精密度优异，可用于实际环境中OCPs准确测定。

**表3:** 5种OCPs在黄河水、农田灌溉水样品中的加标回收率与精密度（*n*=3）

Sample	Analyte	Original/（ng/L）	1.0 ng/L		50 ng/L		100 ng/L	
			Recovery/%	RSD/%	Recovery/%	RSD/%	Recovery/ %	RSD/%
Yellow River water	heptachlor epoxide	ND	102.9	7.7	96.0	4.3	96.6	5.1
	heptachlor	ND	102.3	11.3	95.2	6.0	94.2	2.3
	2，4′-DDD	ND	90.2	6.0	95.5	4.1	96.5	2.8
	2，4′-DDE	ND	89.2	6.7	96.3	8.1	97.4	3.1
	2，4′-DDT	ND	104.0	5.7	103.5	1.7	102.9	3.7
Farmland irrigation water	heptachlor epoxide	ND	97.7	11.3	90.5	7.6	104.8	5.7
	heptachlor	ND	89.0	12.8	102.7	9.7	97.7	2.8
	2，4′-DDD	ND	89.7	7.6	105.5	4.9	98.7	6.4
	2，4′-DDE	ND	104.8	5.4	93.1	7.0	93.1	2.4
	2，4′-DDT	ND	102.9	9.7	97.4	2.6	91.5	6.8

ND： not detected （below LOQ）.

**图9:**
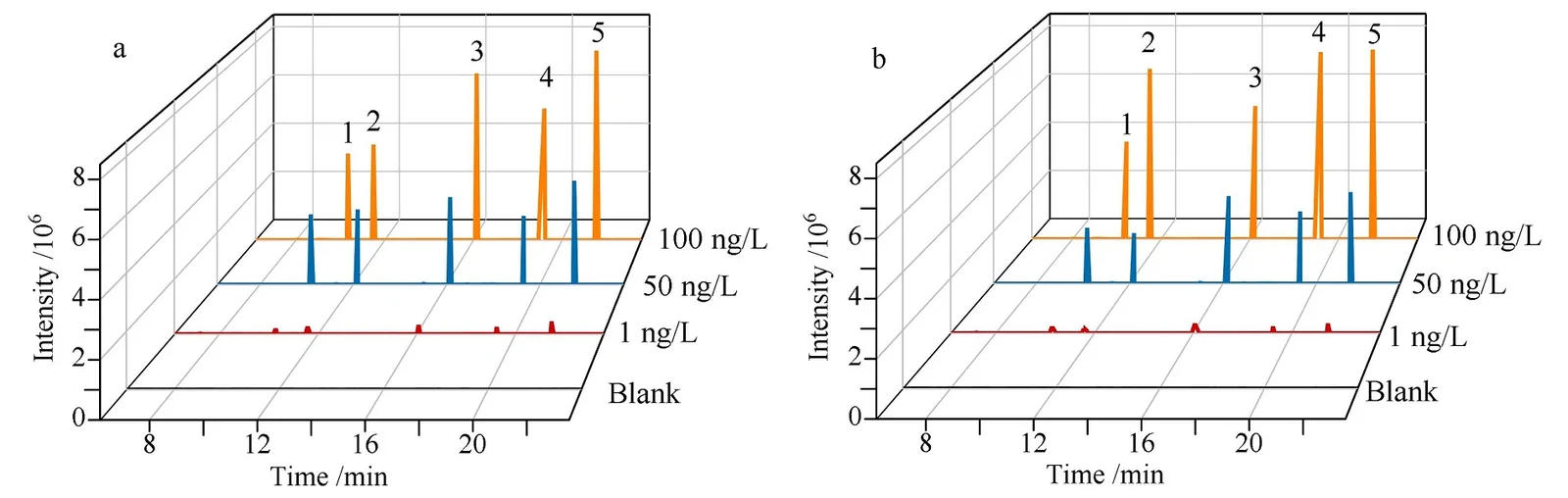
实际水环境样品中5种OCPs的色谱图 a. Yellow River water sample； b. farmland irrigation water sample. 1. heptachlor； 2. heptachlor epoxide； 3. 2，4′-DDE； 4. 2，4′-DDD； 5. 2，4′-DDT.

## 3 结论

本研究采用后合成修饰策略制备氯官能化共价有机骨架Cl-COF作为玻片萃取涂层材料用于μ-SPE，联用GC-ECD建立环境水样中5种痕量OCPs同步检测方法。相较于传统固相萃取小柱，该玻片μ-SPE体系有机溶剂与水样消耗量极低，显著提升样品前处理效率；玻片载体便携易储存，成本低廉，可稳定重复使用30次，适用于野外现场水样快速预处理。Cl-COF依靠*π-π*堆积、疏水作用与卤键多重协同效应特异性吸附目标物，能够有效抵御水体腐殖质与悬浮杂质的基质干扰，方法日内、日间及批间精密度良好，水环境基质适配性优异。该萃取涂层制备简便、结构稳定、富集性能与循环稳定性优异，可满足实际水样中OCPs精准定量检测需求。本研究为功能化COF材料在环境痕量污染物前处理领域的应用提供了新思路，所建立的μ-SPE-GC-ECD检测方法在水环境OCPs痕量筛查与定量分析中具有良好的应用前景。
